# Gender stereotypes and female exercise behavior: mediating roles of psychological needs and negative emotions

**DOI:** 10.3389/fpsyg.2025.1569009

**Published:** 2025-07-08

**Authors:** Kai Guo, Qian Huang

**Affiliations:** ^1^School of Economics and Management, Wuhan Sports University, Wuhan, Hubei, China; ^2^College of Intelligent Sports Engineering, Wuhan Sports University, Wuhan, Hubei, China

**Keywords:** physical activity participation, psychological theory, self-determination theory, sexism in sports, gender stereotypes

## Abstract

**Introduction:**

Integrating Expectancy-Value Theory, Basic Psychological Needs Theory, and Social Identity Theory, this study aims to examine the impact of gender stereotypes on female exercise behavior and to test the chain mediation effects of psychological needs satisfaction in exercise and negative exercise emotions.

**Methods:**

The study uses adapted scales for gender stereotypes, psychological needs satisfaction in exercise, exercise-related emotions, and exercise behavior to survey 790 women in China.

**Results:**

The results show that gender stereotypes are significantly negatively associated with female exercise behavior. Both psychological needs satisfaction in exercise and negative exercise emotions mediate the relationship between gender stereotypes and female exercise behavior. Furthermore, psychological needs satisfaction and negative exercise emotions also jointly play a chain mediating role.

**Discussion:**

Addressing gender stereotypes and fostering environments that support psychological needs fulfillment could improve women’s engagement in exercise. Future interventions should focus on reducing gender bias in sports and promoting inclusive, supportive exercise environments to enhance women’s participation in physical activity.

## 1 Introduction

Physical exercise, as a regular, structured, and repetitive form of physical activity, is a key approach to improving health and preventing chronic diseases. It not only enhances physical fitness and promotes mental well-being, but also strengthens individuals’ capacity for social adaptation, thus holding significant public health value (Eather et al., 2023; Sivaramakrishnan et al., 2024). However, existing studies indicate that participation in physical exercise among adults remains suboptimal globally, with women consistently showing lower levels of engagement than men (Walton et al., 2024). In 2022, approximately 31.3% of adults worldwide did not meet the recommended level of physical activity, with 33.8% of women falling short, compared to 28.7% of men (Strain et al., 2024). This gender disparity cannot be fully explained by biological differences or access to resources, and is more likely influenced by socio-cultural and structural factors. Therefore, investigating the underlying mechanisms that affect women’s participation in physical exercise is not only essential for enhancing their health outcomes, but also crucial for advancing gender equity and achieving universal health goals.

In previous research on the attribution mechanisms and optimization strategies for female exercise behavior, factors such as physical exertion, family exercise environment, socioeconomic status, physical self-concept, and gender differences have been identified as antecedent variables influencing women’s participation in physical exercise (Crossman et al., 2024; Moreno-Llamas et al., 2022; Peng et al., 2023; Schmidt et al., 2015). In recent years, as research has deepened and perspectives broadened, gender stereotypes—culturally embedded and rigid perceptions of male and female behavioral traits—have emerged as a potentially significant yet often overlooked factor influencing female participation in physical exercise (Gentile et al., 2018). Specifically, traditional gender roles tend to frame women within paradigms of softness and modesty while associating strength, competitiveness, and athleticism with masculinity (Hentschel et al., 2019; Plaza et al., 2017). These stereotypes are perpetuated through media narratives, educational settings, and everyday social interactions and are gradually internalized as the belief that physical exercise is inherently a male domain (Rasmussen et al., 2021). Within such a cultural context, women may not only face the risk of being labeled as “masculine” for engaging in sports. However, they may also experience diminished autonomy and a perceived lack of competence in exercise. These psychosocial barriers can ultimately reduce their motivation to participate and lead to behavioral withdrawal. Therefore, uncovering the mechanisms through which gender stereotypes affect female exercise behavior is crucial—not only for addressing health inequities, but also for providing empirical foundations to foster a more gender-inclusive exercise culture.

Once gender stereotypes are formed, they exert a broad influence on both individuals’ psychology and behavior (Anglin et al., 2022). In the context of female physical exercise behavior, such stereotypical perceptions not only directly constrain willingness to participate but may also trigger a chain reaction that undermines the satisfaction of basic psychological needs in exercise and elicits negative exercise-related emotions, thereby forming a systematic mechanism of behavioral inhibition (Sunderji et al., 2024). According to Basic Psychological Needs Theory, three innate psychological needs—autonomy, competence, and relatedness—are fundamental drivers of human motivation and behavior (Deci and Ryan, 2000). When physical exercise is socially constructed as a ‘masculine domain’ due to prevailing gender stereotypes, women may be pressured to relinquish autonomous exercise choices because of perceived incongruence with traditional female roles, may question their competence due to stereotypes of inferior physical ability, or may feel socially excluded in male-dominated exercise environments. The prolonged frustration of these psychological needs not only diminishes intrinsic motivation for exercise but also contributes to the emergence of negative emotional responses such as shame and anxiety, which may further deepen resistance toward participation (Lloyd and Little, 2010). Thus, the fulfillment of basic psychological needs in exercise and negative exercise emotions may serve as important mediating mechanisms linking gender stereotypes to female exercise behavior.

In summary, this study is grounded in the Basic Psychological Needs Theory and aims to address the following core questions: How do gender stereotypes influence female exercise behavior? Do psychological needs satisfaction in exercise and negative exercise emotions serve as mediators in this relationship? Specifically, this study will examine the individual mediating effects of these two variables, as well as their combined mediating effect, to uncover the underlying psychological mechanisms through which gender stereotypes influence female exercise behavior.

## 2 Theoretical foundation and research hypotheses

### 2.1 Gender stereotypes and female exercise behavior

Gender stereotypes refer to the fixed patterns of cognition about the behaviors and personality traits attributed to men and women, which involve the generalization and summarization of the characteristics of the two sexes (Korlat et al., 2022). These stereotypes not only persist and influence social activities but also represent the general expectations people have of male and female members of society (Ellemers, 2018). Such gender role stereotypes serve as a precursor to social gender inequality, which leads to biased gender role expectations (Prentice and Carranza, 2002). Under the influence of these stereotypes, when individuals experience conflicts between their ideal self and actual self (such as diminished self-esteem, restricted personality, or violation), gender role conflicts arise (Good and Sanchez, 2010).

Regarding gender stereotypes, women are stereotypically viewed as possessing communion, meaning they seek relationship maintenance, desire belonging, and emphasize warmth and morality (Loeffler and Greitemeyer, 2023). In contrast, men are stereotypically viewed as possessing agency, meaning they are seen as pursuing goal achievement, taking task-oriented actions, and emphasizing confidence and competence (Hentschel et al., 2019). As a physical practice that reflects the oppositional relationship between the sexes, sports also construct corresponding stereotypes about the body, society, and sports (Plaza et al., 2017; Saemi et al., 2025). These gender differences are often stereotypically understood in exercise behavior, with men being perceived as having better endurance in exercise, while women are thought to possess more stable exercise habits (Themen and van Hooff, 2017). Specifically, in modern society, competitive sports are often associated with masculine traits such as large body size, strong muscles, and agility (Eccles and Harold, 1991). In contrast, women, who are generally smaller in stature, more flexible, and often affected by physiological events such as menstruation, pregnancy, childbirth, and menopause, are perceived as naturally less suited for intense physical sports (Liu et al., 2023; Sunderji et al., 2024). Women’s sports also receive less attention in mass media coverage, which usually focuses more on the appearance or femininity of female athletes than on their athletic performance (Walton et al., 2024). These stereotypes influence women’s participation in sports, as men are more likely to view themselves as stronger and more capable than women, thereby placing greater importance on sports participation (Gentile et al., 2018).

Expectancy-value theory suggests that individuals’ beliefs about their competence, expectations of success, and task value influence their task choice and performance, and that these factors may be influenced by socialization into gender roles (Wigfield and Eccles, 2000). Existing research has shown that women may perceive their athletic abilities as inadequate due to physical or physiological reasons, which can make it difficult for them to engage in physical activities (Peng et al., 2023). Alternatively, they may view exercise as inconsistent with their feminine image of being delicate and quiet, often displaying negative behaviors such as fatigue, social withdrawal, or apathy during exercise (Slater and Tiggemann, 2010). Lindwall and Hassmén (2004) noted that women tend to reduce their willingness to participate in physical activity when they perceive certain sports to be incompatible with their gender identity. This also suggests that gender stereotypes in sports activities have a profound impact on participants’ gender identity and sports behaviors, particularly in the perception of masculine sports, which may have a constraining effect on women’s participation in and behaviors within sports (Sobal and Milgrim, 2019). Therefore, female exercise behavior may be influenced by gender stereotypes in the exercise context, and when women’s exercise behavior contradicts socially accepted gender stereotypes, it may lead to negative consequences for their exercise behavior. Based on this, we propose the following research hypothesis:

*H1*: Gender stereotypes negatively predict female exercise behavior.

### 2.2 The mediating role of psychological needs satisfaction in exercise

Basic Psychological Needs Theory (BPNT) is a branch of Self-Determination Theory and is one of the most widely used frameworks in the study of exercise behavior (Westerskov Dalgas et al., 2024). BPNT posits that humans are born with three basic psychological needs: autonomy, competence, and relatedness (Deci and Ryan, 2000). Autonomy refers to an individual’s desire for their behavior to be freely chosen and decided upon without external control. Competence refers to the desire to experience a sense of mastery when interacting with the social environment. Relatedness refers to the need to feel understood and supported by others, thereby experiencing a sense of belonging (Ryan, 2017). In the context of exercise, psychological needs satisfaction refers to the extent to which the external environment fulfills an individual’s exercise-related psychological needs. It is the essential “nutrient” that supports the internalization of psychological engagement in exercise and the pursuit of active exercise behavior (Mack and Wilson, 2021).

Traditional gender views often categorize women as submissive, gentle, and passive, and women who engage in exercise may conflict with the societal expectations associated with these traits (Chalabaev et al., 2013; Schaillee et al., 2021). When exercise behavior conflicts with gender stereotypes, it may lead women to feel that their exercise choices lack societal respect and recognition, thereby damaging their autonomy in exercise participation and practice (Lloyd and Little, 2010). Additionally, the influence of gender stereotypes may trigger avoidance motivations, limiting opportunities for skill development and mastery of exercise abilities, thereby impacting competence. At the same time, when exercise behavior deviates from gender stereotypes, women may face increased stigma (Peng et al., 2023), such as being labeled as “tomboys,” which can reduce self-esteem and lead to social withdrawal in exercise settings, ultimately preventing the fulfillment of the need for relatedness.

Basic Psychological Needs Theory also suggests that the degree to which individuals internalize external requirements, values, and norms depends on their psychological needs within activities, and the extent of psychological needs satisfaction activates behaviors at varying levels of motivation (Deci and Ryan, 2000) In the promotion of exercise, satisfying psychological needs enhances intrinsic interest and enjoyment in exercise (Westerskov Dalgas et al., 2024), thereby further increasing an individual’s exercise participation (Dunton et al., 2023). Conversely, when an individual’s psychological exercise needs are unmet, they may suffer significant psychological costs, resulting in “negative adaptation,” such as psychological alienation, deprivation, and inefficiency (Gunnell et al., 2013). For women, when their competence, autonomy, and relatedness needs in exercise are not fulfilled, it further strengthens their avoidance motivation toward exercise, negatively impacting their exercise behavior (Lloyd and Little, 2010). In conclusion, society’s gender stereotypes regarding women in sports lead to a lower degree of recognition for women’s participation in physical exercise compared to men. This lack of recognition weakens their satisfaction with psychological exercise needs, further diminishing their engagement in exercise. Based on this, we propose the following research hypothesis:

*H2*: Psychological needs satisfaction in exercise mediates the relationship between gender stereotypes and female exercise behavior.

### 2.3 The mediating role of negative exercise emotions

Negative exercise emotions refer to subjective emotional experiences and cognitive evaluations that are directly related to physical exercise, occurring during or shortly after the exercise period, characterized by distinctly negative features (Lane and Lovejoy, 2001). Compared to males, females experience more anxiety, distress, and lower satisfaction due to traditional societal gender stereotypes (Hively and El-Alayli, 2014). When their exercise behavior contradicts their expectations, it limits their exercise engagement and negatively impacts their psychological adaptation and subsequent behavioral choices (Bevan et al., 2021).

The impact of gender stereotypes on women’s negative exercise emotions primarily manifests in two ways: on one hand, women form complex psychological states, such as cognition, emotion, and self-esteem, based on external support and recognition of their exercise behavior (Laird et al., 2016; Levy and Ebbeck, 2005). If women perceive their exercise behavior as unsupported by society, which contradicts traditional gender stereotypes, they are more likely to experience negative emotions like burnout and anxiety during exercise (He et al., 2024; Hively and El-Alayli, 2014). On the other hand, if their exercise behavior receives more external support, recognition, and attention, the gender role conflict they feel during exercise will be less pronounced, leading to more positive exercise emotions such as confidence and satisfaction (Asztalos et al., 2012).

According to Social Identity Theory, the formation of group stereotypes also follows a psychological process of social comparison (Hornsey, 2008). Gender stereotypes can trigger social comparison in women, diminishing their exercise self-efficacy and positioning them at a disadvantage compared to men, thus reinforcing their negative emotional experiences in exercise contexts (Van Loo et al., 2013). Existing research has shown that the degree of positive or negative emotional change experienced during and after exercise significantly influences subsequent exercise behavior decisions (Bernstein et al., 2019). If participants experience positive emotions during exercise, this can motivate further participation and promote exercise adherence. Conversely, if participants fail to derive satisfaction or positive feelings from exercise, their exercise intentions are likely to decrease, negatively impacting their exercise persistence (Catellier and Yang, 2013; Williams et al., 2008).

In conclusion, when women’s exercise behavior significantly deviates from the long-established gender role expectations shaped by traditional societal gender stereotypes, this deviation may trigger a series of negative exercise emotions. This psychological reaction not only hinders women’s positive attitudes toward exercise but may also further weaken their motivation and actual participation in physical exercise. Based on this, the study proposes the following research hypothesis:

*H3*: Negative exercise emotions mediate the relationship between gender stereotypes and female exercise behavior.

### 2.4 The chain mediating role of psychological needs satisfaction in exercise and negative exercise emotions

Research on individual subjective well-being indicates that individuals can promote positive emotions when their basic psychological needs are met during physical activity, thus enhancing their subjective well-being (Gunnell et al., 2013). Conversely, if these needs are poorly satisfied, negative emotions increase, leading to a decline in well-being (Chang et al., 2015). Sheldon (2012) similarly found that autonomy, competence, and relatedness need to predict positive emotions across different cultures and argued that these needs are rooted in human genetics. When the environment provides sufficient support, the satisfaction of basic psychological needs leads to the emergence of positive emotions (Sheldon, 2012). Specifically, when individuals’ needs are fulfilled, they tend to develop in positive directions, such as self-improvement and self-enhancement (Lin and Chan, 2020). However, such fulfillment is not automatic and requires support and assistance from the external environment (Deci and Ryan, 2008). Existing studies also suggest that when women receive more support from significant others, their motivation, enjoyment, and happiness in exercise increase significantly (Cho et al., 2023).

However, most sports carry traits of masculinity, such as aggressiveness, competitiveness, and adventurousness, which contradict the socially advocated feminine traits. Under the influence of such gender stereotypes, women find it difficult to escape society’s expectations of their gender roles (Ellemers, 2018), and experience conflict between exercise and their gender role (Vieira Sosa et al., 2025). This leads to feelings of being unsupported and unrecognized in their exercise behavior (Patnode et al., 2010). When women’s exercise behavior fails to perceive support from others, their psychological exercise needs remain unmet, further triggering negative psychological reactions (Gunnell et al., 2013).

In conclusion, the presence of gender stereotypes may significantly undermine women’s satisfaction with psychological exercise needs, which, through psychological mechanisms, induces negative exercise emotions. These negative emotions not only affect women’s psychological state but also suppress the persistence and frequency of their exercise behavior. Based on this, the study proposes the following research hypothesis:

*H4*: Psychological needs satisfaction in exercise and negative exercise emotions mediate the relationship between gender stereotypes and female exercise behavior in a chain mediation.

**Figure 1:**
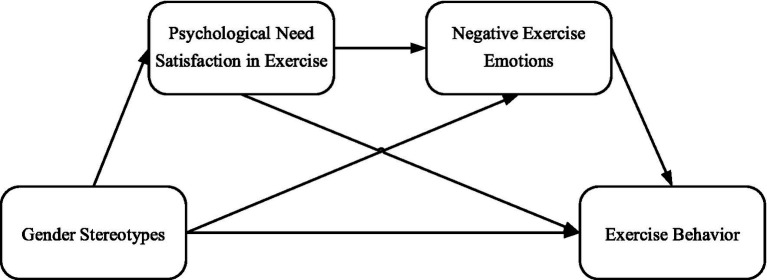
Conceptual model diagram.

In summary, this study constructs a conceptual model of the impact of gender stereotypes, psychological needs satisfaction in exercise, and negative exercise emotions on female exercise behavior. The relationships between the specific variables are shown in Figure 1.

## 3 Research design

### 3.1 Research sample

This study was conducted from March to April 2024, utilizing a combination of offline and online questionnaire surveys targeting the female population. To ensure data quality, screening mechanisms such as attention-check questions and response time controls were incorporated during the questionnaire design phase to eliminate invalid responses. For the offline survey, the research team selected community service centers, educational institutions, and public sports venues in cities across eastern, central, and western provinces of China and distributed 400 questionnaires using convenience sampling. A total of 373 questionnaires were returned, among which 339 were valid after excluding incomplete or invalid responses, resulting in a valid response rate of 90.88%. For the online survey, questionnaires were distributed via the Wenjuanxing platform and disseminated through social media using snowball sampling techniques. A total of 513 responses were collected, with 451 deemed valid after screening, yielding a valid response rate of 87.91%. A total of 790 valid questionnaires were obtained. The sample encompasses a wide geographic area and exhibits considerable diversity in terms of age, educational background, and income level, thereby possessing a certain degree of representativeness. Basic information on the sample is shown in Table 1.

**Table 1:** Basic information of the sample.

Statistical variable	Category	Frequency	Percentage (%)
Gender	Male	0	0
	Female	790	100.0
Age	Under 25 years	147	18.6
	25–35 years	248	31.4
	36–45 years	226	28.6
	Over 45 years	169	21.4
Education level	High school or below	242	30.6
	Associate degree	281	35.6
	Bachelor’s degree	197	24.9
	Master’s degree or above	70	8.9
Monthly income	3,000 or below	235	29.7
	3,001–5,000	288	36.5
	5,001–8,000	154	19.5
	Above 8,000	113	14.3

### 3.2 Measurement tools

#### 3.2.1 Gender stereotypes

Gender stereotypes are culturally embedded cognitive biases, such as the beliefs that women are more emotional and men are more suited to leadership. This study focuses on gender stereotypes in the sports domain, given its prominent role in gender role socialization and its suitability for examining the effects of stereotypes within a specific situational context. The scale was adapted from relevant work by Sage and Loudermilk (1979), Huang and Zhang (2025), and Schmader et al. (2004), and refined to suit the current study (Huang and Zhang, 2025; Sage and Loudermilk, 1979; Schmader et al., 2004). Seven items were selected, such as “Social expectations of women’s participation in sports are much lower than men’s,” which were rated on a 7-point Likert scale (1 for “strongly disagree,” 7 for “strongly agree”), with higher scores indicating higher levels of stereotyping. In this study, the Cronbach’s α coefficient for this scale was 0.946.

#### 3.2.2 Exercise behavior

In this study, “physical exercise” is defined as planned, structured physical activity undertaken during leisure time with a certain level of intensity, aimed at improving or maintaining physical health and fitness. Participants were asked to report activities including, but not limited to, jogging, running, aerobics, yoga, swimming, cycling, strength training, badminton, tennis, and basketball. Routine physical activities such as commuting or household chores were excluded from the analysis. This study employed the revised Physical Activity Rating Scale-3 (PARS-3), developed by Liang (1994), which has been widely used to assess physical exercise behavior in the Chinese context (Liang, 1994). This scale uses a 5-point Likert scale, with scores ranging from 1 to 5. According to the evaluation criteria, the exercise load score is calculated as: intensity × (duration - 1) × frequency, with a maximum score of 100 and a minimum score of 0, representing exercise behavior. In this study, the Cronbach’s α coefficient for this scale was 0.880.

#### 3.2.3 Psychological needs satisfaction in exercise

The measurement of psychological needs satisfaction in exercise is represented by the Psychological Needs Satisfaction in Exercise Scale (PNSE) developed by Wilson et al. (2006). This scale includes three dimensions: competence needs, autonomy needs, and relatedness needs. The scale is divided into these three dimensions, each containing 6 items, for a total of 18 items. The competence dimension includes items such as “I feel confident in my ability to complete challenging exercise tasks”; the autonomy dimension includes items such as “I can freely choose the exercise activities I want to engage in”; and the relatedness dimension includes items such as “In exercise, I feel a good connection and interaction with those I exercise with.” The items are rated on a 7-point Likert scale, where 1 represents “strongly disagree” and 7 represents “strongly agree,” with higher scores indicating greater satisfaction of psychological needs in exercise. In this study, the Cronbach’s α values for each dimension are 0.929, 0.947, and 0.947, respectively, with a total Cronbach’s α of 0.950, indicating a high level of internal consistency and reliability of the scale.

#### 3.2.4 Negative exercise emotions

The measurement of negative exercise emotions was adapted from the negative exercise emotion subscale of the Physical Activity Affect Scale developed by Lox et al. (2000) (Lox et al., 2000), this study made appropriate modifications to the original scale to better fit the context of this research. The specific modifications involved adjusting the language of certain items to more closely align with the emotional experiences of female participants during exercise. The final modified scale consists of four items, such as: “I feel that exercise is a burden to me, rather than an enjoyment.” The items are rated on a 7-point Likert scale, where 1 represents “strongly disagree” and 7 represents “strongly agree,” with higher scores indicating higher levels of negative exercise emotions in women. In this study, the Cronbach’s α for this scale was 0.916.

### 3.3 Data analysis methods

This study is a cross-sectional questionnaire survey designed to explore the influencing factors of female exercise behavior. Data analysis was conducted using SPSS 22.0 and AMOS 24.0. SPSS 22.0 was used for data entry, descriptive statistics, and correlation analysis, as well as for conducting hierarchical regression analyses to examine the direct effects among variables and the preliminary role of mediators. Additionally, AMOS 24.0 was employed to perform Confirmatory Factor Analysis (CFA) and Structural Equation Modeling (SEM). The significance of the chained mediation effect was tested using the Bootstrap method with 5,000 resamples. While hierarchical regression analysis focuses on decomposing local relationships among variables, structural equation modeling (SEM) combined with the Bootstrap method validates complex pathways in terms of overall model fit and statistical robustness. These two analytical approaches complement each other, thereby ensuring the reliability of the analysis results.

## 4 Results and analysis

### 4.1 Control and testing of common method bias

This study employed anonymous responses and mitigated common method bias through the use of reverse-coded items and mixed item ordering. However, since data were collected via self-reports, some degree of common method bias was unavoidable. For this reason, all question items were tested using the Harman’s single-factor test. The results indicated that there were 6 factors with eigenvalues greater than 1, and the first unrotated factor accounted for 37.613% of the variance, which is below the 40% threshold. This suggests that the common method bias is acceptable and does not significantly affect the validity of subsequent analyses.

### 4.2 Confirmatory factor analysis

This study conducted confirmatory factor analysis (CFA) using AMOS 24.0 software. The results indicated that the measurement model demonstrated a good fit: χ^2^/df = 1.443, RMR = 0.080, GFI = 0.952, AGFI = 0.941, NFI = 0.974, TLI = 0.991, CFI = 0.992, RMSEA = 0.024. As shown in Table 2, all factor loadings for the items were greater than 0.6, and the composite reliabilities (CR) for all variables exceeded the recommended threshold of 0.7. Additionally, the average variance extracted (AVE) was also higher than the recommended level of 0.50, indicating that the scale demonstrates good convergent validity. As presented in Table 3, the square roots of all AVEs were greater than the correlations between the corresponding variables, confirming that the scale possesses good discriminant validity.

**Table 2:** Questionnaire items.

Variable	Item	Load	SD	AVE	CR
Gender stereotypes	GS1	0.857	0.186	0.740	0.952
	GS2	0.856			
	GS3	0.858			
	GS4	0.887			
	GS5	0.889			
	GS6	0.846			
	GS7	0.828			
Competence	COM1	0.839	0.180	0.684	0.928
	COM2	0.856			
	COM3	0.871			
	COM4	0.813			
	COM5	0.775			
	COM6	0.804			
Autonomy	AUT1	0.878	0.181	0.751	0.948
	AUT2	0.866			
	AUT3	0.871			
	AUT4	0.876			
	AUT5	0.859			
	AUT6	0.850			
Relatedness	REL1	0.847	0.161	0.746	0.946
	REL2	0.853			
	REL3	0.884			
	REL4	0.874			
	REL5	0.854			
	REL6	0.868			
Negative exercise emotions	NEE1	0.853	0.164	0.775	0.932
	NEE2	0.887			
	NEE3	0.930			
	NEE4	0.849			
Exercise behavior	EB1	0.865	0.103	0.713	0.882
	EB2	0.878			
	EB3	0.788			

**Table 3:** Correlation coefficients of latent variables.

Variables	GS	COM	AUT	REL	NEE	EB
GS	0.860					
COM	−0.173	0.827				
AUT	−0.155	0.539	0.867			
REL	−0.140	0.509	0.678	0.864		
NEE	0.505	−0.194	−0.178	−0.178	0.880	
EB	−0.340	0.519	0.467	0.419	−0.295	0.844

The diagonal data of the matrix represent the square root of the AVE values, and the lower half of the matrix represents the correlation coefficient.

### 4.3 Descriptive statistics and correlation analysis

To explore the relationships among gender stereotypes, psychological needs satisfaction in exercise, negative exercise emotions, and exercise behavior, statistical and correlation analyses were conducted on these research variables. The results are shown in Table 4. Gender stereotypes were significantly negatively correlated with psychological needs satisfaction in exercise (β = −0.229, *p* < 0.01) and exercise behavior (β = −0.437, *p* < 0.01), and significantly positively correlated with negative exercise emotions (β = 0.497, *p* < 0.01). Psychological needs satisfaction in exercise was significantly negatively correlated with negative exercise emotions (β = −0.298, *p* < 0.01) and significantly positively correlated with exercise behavior (β = 0.516, *p* < 0.01). Negative exercise emotions were significantly negatively correlated with exercise behavior (β = −0.462, *p* < 0.01). The significant correlations among the variables align with the direction of the research hypotheses, providing a necessary foundation for subsequent model analysis.

**Table 4:** Results of descriptive statistics and correlation analysis.

Variables	*M*	SD	GS	PESE	NEE	EB
GS	4.454	1.700	1			
PESE	4.668	1.374	−0.229**	1		
NEE	4.214	1.713	0.497**	−0.298**	1	
EB	33.172	29.721	−0.437**	0.516**	−0.462**	1

**p < 0.01; PESE, Psychological Needs Satisfaction in Exercise.

### 4.4 Regression model testing

Hierarchical regression analysis was used to establish the regression models, with the results presented in Table 5. Model 3 shows that the regression coefficient for gender stereotypes on female exercise behavior is −0.437 (*p* < 0.001), indicating that the level of gender stereotypes negatively predicts female exercise behavior, supporting Hypothesis H1. Models 1 and 2 show that the regression coefficients for gender stereotypes on female psychological needs satisfaction in exercise and negative exercise emotions are −0.189 (*p* < 0.001) and 0.499 (*p* < 0.001), respectively, indicating that the level of gender stereotypes negatively predicts psychological needs satisfaction in exercise and positively predicts negative exercise emotions in women. Models 4 and 5 show that the regression coefficients for psychological needs satisfaction in exercise and negative exercise emotions on female exercise behavior are 0.521 (*p* < 0.001) and −0.462 (*p* < 0.001), respectively, suggesting that psychological needs satisfaction positively predicts female exercise behavior, while negative exercise emotions negatively predict it. Additionally, Models 6, 7, and 8 show that when the variables of psychological needs satisfaction and negative exercise emotions are introduced separately or together, although gender stereotypes still predict female exercise behavior, the strength of the relationship weakens. This suggests that psychological needs satisfaction and negative exercise emotions may mediate the relationship between gender stereotypes and female exercise behavior, providing essential support for the subsequent mediation effect testing.

**Table 5:** Results of regression analysis.

Variables	PESE	NEE	EB					
	Model 1	Model 2	Model 3	Model 4	Model 5	Model 6	Model 7	Model 8
Age	0.032	−0.026	0.002	−0.015	−0.013	−0.009	−0.007	−0.013
Education level	−0.067	−0.052	0.011	0.032	−0.014	0.031	−0.006	0.017
Monthly income	−0.072	0.029	0.006	0.058	0.036	0.029	0.016	0.034
GS	−0.189***	0.499***	−0.437***			−0.333***	−0.274***	−0.230***
PESE				0.521***		0.443***		0.399***
NEE					−0.462***		−0.326***	−0.228***
*R* ^2^	0.059	0.250	0.438	0.522	0.215	0.643	0.271	0.413
Adj. *R*^2^	0.054	0.246	0.191	0.272	0.211	0.413	0.266	0.409
*F*	12.344***	65.487***	46.465***	73.364***	53.657***	91.898***	58.268***	91.898***

*p < 0.05; **p < 0.01; ***p < 0.001.

### 4.5 Mediation effect testing

**Figure 2:**
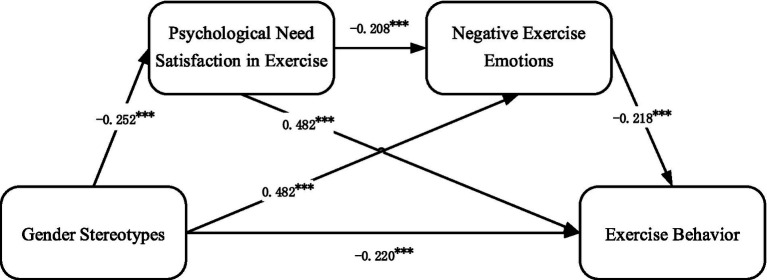
Conceptual model main path coefficient diagram. *p < 0.05; **p < 0.01; ***p < 0.001.

A structural equation model was used to establish the relationship model with gender stereotypes as the independent variable, exercise behavior as the dependent variable, and psychological needs satisfaction in exercise and negative exercise emotions as mediating variables (see Figure 2). The model fit statistics are as follows: χ^2^/df = 1.611, RMR = 0.127, GFI = 0.947, AGFI = 0.935, NFI = 0.969, TLI = 0.986, CFI = 0.988, RMSEA = 0.028. These statistics indicate that the measurement model has good fit. As shown in Figure 2, gender stereotypes significantly negatively affect exercise behavior (β = −0.220, *p* < 0.001), further confirming Hypothesis H1. Additionally, gender stereotypes significantly negatively affect psychological needs satisfaction in exercise (β = −0.252, *p* < 0.001) and significantly positively affect negative exercise emotions (β = 0.482, *p* < 0.001). Psychological needs satisfaction in exercise significantly negatively affects negative exercise emotions (β = −0.208, *p* < 0.001) and significantly positively affects exercise behavior (β = 0.482, *p* < 0.001). Negative exercise emotions significantly negatively affect exercise behavior (β = −0.218, *p* < 0.001).

To verify the mediating effects of psychological needs satisfaction in exercise and negative exercise emotions in the path through which gender stereotypes affect female exercise behavior, this study used the Bootstrap method for significance testing of the mediation effects, employing AMOS 24.0 software. A total of 5,000 bootstrap resamples were conducted on 790 samples, and if the 95% confidence interval does not contain 0, it indicates the presence of a mediation effect. The Bootstrap analysis for the significance testing of the mediation effects in this study is shown in Table 4.

As shown in Table 6, psychological needs satisfaction in exercise and negative exercise emotions exert significant mediating effects in the relationship between gender stereotypes and exercise behavior. The total indirect effect was β = −0.188 (95% CI [−0.240, −0.141], *p* < 0.001), accounting for 52.08% of the total effect. Specifically, gender stereotypes indirectly influenced exercise behavior through psychological needs satisfaction in exercise, with a standardized indirect effect of β = −0.096 (95% CI [−0.133, −0.066], *p* < 0.001), accounting for 26.59% of the total effect. This indicates that the mediation pathway is significant, supporting Hypothesis H2. In addition, gender stereotypes also indirectly influenced exercise behavior through negative exercise emotions, with a standardized indirect effect of β = −0.083 (95% CI [−0.119, −0.050], *p* < 0.001), accounting for 22.99% of the total effect, thereby supporting Hypothesis H3. Furthermore, gender stereotypes influenced exercise behavior indirectly through a sequential chain of psychological needs satisfaction in exercise followed by negative exercise emotions, with an effect size of β = −0.009 (95% CI [−0.015, −0.005], *p* < 0.001), accounting for 2.49% of the total effect. This chain mediation path was also significant, providing support for Hypothesis H4.

**Table 6:** Mediating effects test.

Path	Effect value	SE	*p*	Bias-corrected 95% CI		Percentage (%)
				Lower	Upper	
Total effect	−0.361	0.027	***	−0.413	−0.308	100
Direct effect	−0.173	0.033	***	−0.240	−0.109	47.92
Indirect effects	−0.188	0.025	***	−0.240	−0.141	52.08
GS → PESE→EB	−0.096	0.017	***	−0.133	−0.066	26.59
GS → NEE → EB	−0.083	0.018	***	−0.119	−0.050	22.99
GS → PESE→NEE → EB	−0.009	0.002	***	−0.015	−0.005	2.49

***p < 0.001.

## 5 Discussion

### 5.1 The impact of gender stereotypes on female exercise behavior

The results of this study indicate that gender stereotypes significantly negatively predict female exercise behavior, showing that women who hold higher levels of gender stereotypes in sports tend to have a noticeable decline in their exercise participation. This result is somewhat consistent with previous research, which has generally focused on the overall impact of gender stereotypes on sports performance and participation, emphasizing how psychological and cultural factors influence gender differences in sports (Chalabaev et al., 2013). However, this study further explores the specific mechanisms by which gender stereotypes influence women’s exercise behavior. Building on previous research, this study suggests that the negative impact of gender stereotypes on female exercise behavior can be explored from both social-cultural and individual psychological perspectives.

First, from a social-cultural perspective, traditional social structures generally favor the allocation of sports resources and power to men, shaping biases and discrimination against women’s participation in sports (Evans and Pfister, 2021). Influenced and reinforced by traditional gender culture and societal gender role expectations, some women unconsciously internalize these as gender role stereotypes, thereby developing a value-based understanding of societal gender roles (Korlat et al., 2022). If women align with these gender values, they may develop a psychological rejection of sports, which leads to a natural disregard, devaluation, or even denial of physical exercise and its value (He et al., 2024). From an individual psychological perspective, emotion and memory theory highlights that an individual’s memory system stores emotional experiences associated with task completion (Tyng et al., 2017). When women face social evaluations, their cognitive memories of past exercise behaviors may shape their beliefs, causing them to view delicate, gentle, and calm behavior as more socially acceptable than the active, agile, and strong attributes associated with sports. Furthermore, to maintain self-esteem and avoid being stigmatized as “tomboys” or “unfeminine,” women may display lower levels of activity and persistence in exercise.

In summary, under the influence of gender stereotypes in society, women are more likely to adopt a “socially acceptable” mindset in exercise to gain approval from others. This psychological tendency undoubtedly has a significant negative impact on women’s exercise behavior.

### 5.2 The mediating role of psychological needs satisfaction in exercise

The results of this study indicate that psychological needs satisfaction in exercise mediates the negative impact of gender stereotypes on female exercise behavior: stereotypes reduce this satisfaction, which in turn suppresses exercise participation. Previous studies have shown that when basic psychological needs are satisfied, individuals tend to engage more actively with their environment and participate in behaviors associated with positive outcomes. Conversely, when basic psychological needs are thwarted or unmet, individuals endure significant psychological costs and are more prone to “negative adaptation” behaviors (Vansteenkiste et al., 2020). For example, Orkibi and Ronen (2017) found that the satisfaction of basic psychological needs significantly positively predicted prosocial behaviors and well-being (Orkibi and Ronen, 2017). Yan et al. (2024) demonstrated that when adolescents possess positive exercise cognition and their basic psychological needs (such as autonomy, competence, and relatedness) are satisfied, they are more likely to engage in regular exercise behavior (Yan et al., 2024).

In line with these findings, this study also reveals that the satisfaction of psychological needs in exercise significantly promotes women’s exercise behavior. However, this study goes a step further by not only focusing on the effect of needs satisfaction itself but also tracing its origins. It clearly identifies gender stereotypes—as an external sociocultural structure—as a key factor undermining women’s ability to satisfy their psychological needs. This finding also supports self-determination theory, which posits that basic psychological needs mediate the development between external environments and individuals’ internal growth (Deci and Ryan, 2000). Any event that prevents the fulfillment of these needs generates negative effects, leading to a decline in internal motivation and a decrease in behavioral initiative (Dysvik et al., 2013). For instance, social stigma, as an external pressure mechanism, triggers secondary evaluations by individuals, negatively impacting their exercise motivation through the satisfaction of psychological needs (Liu et al., 2021). On the contrary, Shannon et al. (2020) noted that mindfulness, as an internal support mechanism, can enhance individuals’ self-regulation abilities and satisfaction of basic psychological needs, thereby reducing stress and improving well-being (Shannon et al., 2020). In this study, however, gender stereotypes hinder the satisfaction of women’s psychological needs in exercise, leading to negative effects on subsequent exercise behavior.

Specifically, gender stereotypes lead women to subconsciously perceive sports as more aligned with men, while the self is expected to remain quiet and composed (Hermann and Vollmeyer, 2016; Spence and Sawin, 2014). This belief causes women to overlook their personal growth and development in exercise, reducing the likelihood of actively engaging in physical activity and failing to satisfy their autonomy needs. Additionally, they are less willing to challenge moderate or high-difficulty sports, thus failing to meet their competence needs. They also struggle to form good relationships with others during exercise, preventing the satisfaction of their relatedness needs. When women’s psychological exercise needs are unmet, they develop avoidance coping strategies, leading to a decrease in exercise behavior.

### 5.3 The mediating role of negative exercise emotions

The results of this study indicate that negative exercise emotions significantly mediate the relationship between gender stereotypes and female exercise behavior, suggesting that gender stereotypes can indirectly suppress women’s exercise behavior by triggering negative exercise emotions. This is consistent with the emotional pathways of studies that have explored the negative impact of general negative life events on physical activity behavior by inducing negative workout emotions in individuals (Bourke et al., 2021; Lucibello et al., 2023). However, the present study focuses on the unique influence of a specific socio-cultural antecedent variable, “gender stereotypes,” on negative exercise emotions and behaviors in the female population, and reveals pathways that differ from those of general stressors. This study proposes that the mediating effect of negative exercise emotions between gender stereotypes and female exercise behavior operates through three main mechanisms:

First, from a socio-psychological perspective on exercise, a positive leisure exercise environment promotes feelings of relaxation and comfort, effectively reducing negative exercise emotions and enhancing exercise behavior (Li, 2024). However, under the influence of gender stereotypes, current society generally fosters the view that sports represent a male-oriented display of athleticism (Plaza et al., 2017), with women typically expected to play a supportive role in men’s sports. This exercise environment somewhat suppresses women’s enthusiasm for participating in physical exercise, affecting their perceptions and attitudes toward it.

Second, from the perspective of self-worth and cognition, when women experience intense gender role conflicts during exercise, they receive significant information related to adversity and frustration (Myre et al., 2023). The pervasive gender stereotypes transmitted by society can alter women’s cognitive structures around exercise, making their understanding of its value and significance increasingly narrow, unclear, and irrational. As a result, they become more likely to exhibit negative, disengaged, and aimless behaviors during exercise, with their participation becoming more disorganized, tedious, and unsustainable.

Third, according to the Expectancy-Value Theory, gender stereotypes in the cultural environment create a bias between the ideal self and the real self for women in the context of exercise (Eccles and Harold, 1991). When this discrepancy between reality and societal expectations occurs, it often leads to strong feelings of inadequacy and other negative emotions. Under the influence of psychological defense mechanisms, women may reduce their participation in exercise as a way of coping with the self-deprecating emotional reactions caused by internalizing external negative evaluations during exercise.

### 5.4 The chain mediating role of psychological needs satisfaction in exercise and negative exercise emotions

The results of the mediation effect testing in this study indicate that psychological needs satisfaction in exercise and negative exercise emotions have a mediating effect on the chain between gender stereotypes and female exercise behavior. Although the effect size of the chain mediation path is relatively small (standardized effect = −0.009, 2.49% of the total effect), its confidence interval does not include zero, indicating statistical significance. Theoretically, it reveals how gender stereotypes affect women’s exercise behavior through a layered psychological process—from reduced motivation to increased emotional exhaustion—gradually weakening their willingness to participate in physical activity. The study found that psychological needs satisfaction significantly negatively predicts negative exercise emotions, which is consistent with previous research (Gunnell et al., 2013). For example, a specialized survey of athletes revealed that when athletes’ basic psychological needs are effectively met during exercise, they tend to display more positive emotional experiences, and this state of satisfaction shows a significant negative correlation with exercise burnout and negative emotions (Bartholomew et al., 2011).

Moreover, previous research has thoroughly confirmed that when individuals perceive support from others, it not only significantly enhances their intrinsic motivation for engaging in physical exercise but also actively promotes the optimization and enhancement of their psychological functions (Stevens et al., 2020). Specifically, this social support helps meet individuals’ basic psychological needs, such as autonomy, competence, and relatedness, during exercise, which significantly boosts their psychological satisfaction with exercise behavior and overall psychological well-being. In studies examining gender differences in sports, it has been noted that, compared to men, women face stronger social comparisons regarding physical appearance and athletic performance (Ferguson et al., 2015), and their motivation to maintain positive psychological health outcomes tends to be shorter-lived (Vlachopoulos, 2008), with more negative self-evaluations (Adam et al., 2021). When gender roles contradict traditional societal gender stereotypes, women feel that their exercise behavior is not recognized or supported by others, which reduces their psychological needs satisfaction in exercise.

However, psychological needs satisfaction is one of the internal motivational systems driving women to engage in physical exercise, and it affects the psychological processes related to the development of their exercise emotions (Lloyd and Little, 2010). Therefore, when psychological exercise needs are unmet, they intensify women’s negative exercise emotional experiences, making it difficult for women to actively engage in exercise behavior. From this, it can be concluded that psychological needs satisfaction in exercise and negative exercise emotions are the continuous links that connect gender stereotypes to female exercise behavior.

## 6 Conclusions and future directions

### 6.1 Conclusions and implications

By constructing a structural equation model, this study systematically reveals, both theoretically and empirically, the negative impact of gender stereotypes on women’s exercise behavior and its underlying psychological mechanisms. The findings indicate that gender stereotypes not only directly suppress women’s exercise behavior, but also indirectly weaken it through a chain-mediated pathway involving psychological needs satisfaction in exercise and negative exercise emotions. Furthermore, the study confirms that satisfaction of psychological needs in exercise negatively predicts negative exercise emotions and positively predicts exercise behavior, while negative exercise emotions significantly inhibit women’s participation in physical activity.

These findings contribute to theory in two main aspects. First, the study breaks through the limitations of previous research, which have focused on physiological differences, socioeconomic status, or family role distribution as the primary factors influencing women’s exercise behavior. By introducing gender stereotypes as a socio-psychological dimension, the study enriches the theoretical framework of exercise behavior research. Second, by clarifying the mediating mechanisms of psychological needs satisfaction in exercise and the negative emotions associated with exercise, the study expands the application of basic psychological needs theory and emotional experience within exercise contexts, offering new explanatory pathways for understanding individual exercise decision-making under socio-cultural influences.

Based on the above conclusions, the study offers the following recommendations to promote women’s participation in physical activity. First, society should actively dismantle gender stereotypes surrounding women’s involvement in sports and build a gender-friendly exercise culture. Since sports themselves are not inherently gendered, the public should discard gender-labeling perceptions of sports and enhance women’s sense of social recognition and belonging in exercise contexts. Second, it is crucial to advocate for and implement “androgynous education,” encouraging women to transcend stereotypical gender roles and develop a flexible and multifaceted self-concept, thereby boosting their psychological autonomy and motivation in exercise participation. Third, sports organizations and businesses should pay attention to the personalized needs of women by developing exercise programs and services that align with their physical characteristics and personal interests, thereby enhancing their sense of competence, belonging, and willingness to participate in the long term. Finally, to alleviate potential negative emotions that may arise during exercise, it is essential to strengthen psychological support resources in sports, providing women with professional services in emotional regulation and counseling, which will further promote stable and healthy exercise behavior.

## 7 Limitations and future directions

The limitations of this study are as follows, and these limitations also provide directions for future research: (1) This study employed self-reported questionnaires to collect data. Although reverse-coded items and anonymous responses were used to reduce common method bias, the data may still be influenced by social desirability effects and subjective errors, potentially affecting the conclusions. Future research may consider integrating multiple data sources, such as behavioral tracking, third-party evaluations, or laboratory measurements, to enhance the objectivity and reliability of the data. (2) Due to the cross-sectional design of this study, causal relationships and long-term effects among variables cannot be determined. Future studies could adopt longitudinal surveys or experimental designs to further explore the dynamic effects of gender stereotypes on women’s physical activity. (3) The mediating mechanisms between gender stereotypes and female exercise behavior, aside from psychological need satisfaction in exercise and negative exercise emotions, may also include other factors, such as exercise commitment, self-efficacy, and stress perception, all of which have been shown to mediate the relationship between social cognition and exercise behavior. Future research should further consider a broader range of variables to provide comprehensive insights and references for understanding the antecedent mechanisms of female exercise behavior. (4) Given the constraints on questionnaire length and concerns over participant privacy, key control variables such as body mass index (BMI) and occupation were not included in the present study, which may have limited the explanatory power of the model. Future research could incorporate these variables under more comprehensive data collection conditions to enhance the robustness and generalizability of the findings. (5) Although this study focused on context-specific stereotypes, it did not address broader societal gender stereotypes. This may limit a more comprehensive understanding of gender stereotypes as a general sociopsychological mechanism. Future research could consider employing more inclusive measures of gender stereotypes.

## Data Availability

The original contributions presented in the study are included in the article/supplementary material, further inquiries can be directed to the corresponding author.
